# Aberrant *S100A16* expression might be an independent prognostic indicator of unfavorable survival in non-small cell lung adenocarcinoma

**DOI:** 10.1371/journal.pone.0197402

**Published:** 2018-05-10

**Authors:** De Chen, Linjie Luo, Chao Liang

**Affiliations:** Department of Respiratory Medicine, the First People's Hospital of Yibin, Yibin, China; University of Nebraska Medical Center, UNITED STATES

## Abstract

S100A16 is a conserved member of the S100 protein family in mammals. Its upregulation was observed in many tumors and is related to malignant transformation. In this study, we explored the independent prognostic value of *S100A16* in terms of overall survival (OS) and recurrence-free survival (RFS) by performing a retrospective study, using data in The Cancer Genome Atlas (TCGA)-lung adenocarcinoma (LUAD). Besides, by using deep sequencing data in TCGA-LUAD, we also explored the association between *S100A16* expression and its DNA methylation and copy number alterations (CNAs). Results showed that the primary LUAD tissues (N = 514) had significantly elevated *S100A16* expression compared with the normal lung tissues (N = 59). Based on OS data of 502 primary LUAD cases, we found that high *S100A16* expression was correlated with inferior OS. The following univariate and multivariate analysis confirmed that increased *S100A16* expression was an independent prognostic indicator of unfavorable OS (HR: 1.197, 95%CI: 1.050–1.364, *p* = 0.007) and RFS (HR: 1.206, 95%CI: 1.045–1.393, *p* = 0.011). By examining the DNA methylation data in TCGA-LUAD, we found that some *S100A16* DNA CpG sites were generally hypermethylated in normal tissues, but not in LUAD tissues. Regression analysis identified a moderately negative correlation between *S100A16* expression and its DNA methylation. In comparison, although DNA amplification (+1/+2) was frequent (378/511, 74%) in LUAD patients, it was not associated with increased *S100A16* expression. Based on findings above, we infer that aberrant *S100A16* expression might be modulated by its DNA hypomethylation and serves as an independent prognostic indicator of unfavorable OS and RFS in LUAD.

## Introduction

The S100 protein family contains over 25 calcium-binding proteins with the EF-hand motif. These family members are implicated in some critical intracellular and extracellular functions, including cell proliferation, apoptosis, cell invasion and motility, cytoskeleton interactions, regulation of transcriptional factors, autoimmunity, chemotaxis, inflammation and pluripotency [1].

S100A16 is a conserved member of the S100 protein family in mammals. Its upregulation was observed in many tumors and is related to malignant transformation [2]. In breast cancer, S100A16 upregulation could promote epithelial-mesenchymal transition (EMT) via the Notch1 pathway, thereby enhancing cancer cell invasion [3, 4]. In prostate cancer, S100A16 could promote cell proliferation and metastasis via AKT and ERK cell signaling pathways [5]. In colorectal cancer, membrane S100A16 expression might serve as an independent prognostic marker for overall survival (OS) [6]. Two recent studies (N = 170 and N = 65 respectively) observed that *S100A16* is significantly upregulated in lung adenocarcinoma (LUAD) and might be an independent prognostic indicator of poor OS [7, 8]. However, the small sample size limits their statistical power.

The Cancer Genome Atlas (TCGA), supervised by the National Cancer Institute's Center for Cancer Genomics and the National Human Genome Research Institute is a large project, which provides a reliable pool of molecular, clinicopathological and survival data sets of over 30 cancers [9]. The genetic and clinicopathological data of over 500 patients with primary LUAD were recorded in TCGA-LUAD [10]. In this study, we further explored the independent prognostic value of *S100A16* in terms of OS and recurrence-free survival (RFS) by performing a retrospective study, using data in TCGA-LUAD.

Although aberrant *S100A16* expression was observed in LUAD, the mechanisms of its dysregulation are far from being fully understood. One previous study found that S100A14 could interact with S100A16 and increase its expression via post-transcriptional regulation [11]. In this study, by using deep sequencing data in TCGA-LUAD, we also explored the association between *S100A16* expression and its DNA methylation and copy number alterations (CNAs).

## Materials and methods

### Secondary analysis using data in TCGA-LUAD

Data screening in TCGA-LUAD was performed by using the UCSC Xena Browser (https://xenabrowser.net/). In this database, we found that 514 primary LUAD had gene expression measured by RNA-seq and 502 primary LUAD cases had RNA-seq and intact OS data recorded at the same time. The clinicopathological data, including age at initial pathologic diagnosis, gender, smoking history, pathologic stage, nodal status, the status of residual tumors, the history of radiation therapy and targeted molecular therapy, recurrence status, RFS in days, OS status and OS in days were downloaded for secondary analysis, as showed in S1 Table. The DNA methylation data (Illumina 450k infinium methylation beadchip) and the gene-level thresholded GISTIC2-processed CNAs data were downloaded to explore the potential mechanisms of *S100A16* dysregulation in LUAD.

### Data mining in the Cancer Cell Line Encyclopedia (CCLE)

*S100A16* expression and its DNA methyaltion in LUAD cell lines were examiend by using data from the CCLE, which provides public access to genomic data (including Copy Number, mRNA expression (Affy), RPPA, RRBS, and mRNA expression (RNAseq)), analysis and visualization for over 1100 cell lines [12].

### Immunohistochemistry (IHC) staining

*S100A16* expression at the protein level in normal lung tissues and in LUAD tissues was examined using IHC staining data in the Human Protein Atlas (HPA) (http://www.proteinatlas.org/) [13–15].

### Data mining in the Kaplan-Meier Plotter

The association between *S100A16* expression and OS or first-progression free survival (FPS) in LUAD patients was also examined by data mining in the Kaplan-Meier Plotter, an online survival analysis software to assess the prognostic value of biomarkers using transcriptomic data in non-small-cell lung cancer (NSCLC) [16]. Pooled results based on previous available genomic studies were generated. OS and FPS curves were generated by setting median *S100A16* expression as the cutoff. The hazard ratio (HR) with 95% confidence intervals (CI) and log-rank *p*-value were calculated.

### Statistical analysis

Data were presented as means and standard deviations (SDs). Data analysis was performed by using GraphPad Prism 6.0 (GraphPad Inc.) or SPSS 19.0 software package (SPSS Inc.). *S100A16* expression between the groups with different clinicopathological parameters were compared using Welch’s t-test. *χ*^*2*^ tests were used to assess the association between *S100A16* expression and the clinicopathological parameters. Kaplan-Meier curves of OS and RFS (data from TCGA-LUAD) were generated by using GraphPad Prism 6.0, by setting the median *S100A16* expression as the cutoff. Log-rank test was conducted to assess the significance of the difference between the survival curves. Univariate and multivariate Cox regression analysis was performed to determine the independent prognostic value of *S100A16* expression (as a continuous variable) in terms of OS and RFS. Regression analysis was performed to assess the correlation between *S100A16* expression and its DNA methylation. *p*<0.05 was considered statistically significant.

## Results

### LUAD tissues had significantly elevated *S100A16* expression compared with normal lung tissues

**Fig 1:**
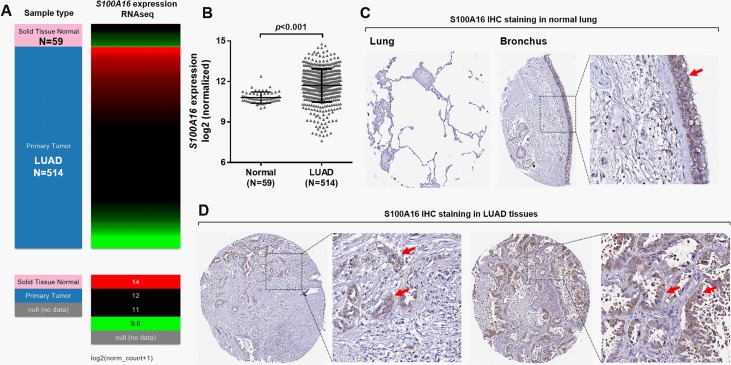
LUAD tissues had significantly elevated *S100A16* expression compared with normal lung tissues. **A-B.** Heatmap (A) and plots chart (B) showing *S100A16* RNA expression in LUAD tissues (N = 514) and in normal lung tissues (N = 59). **C-D.** S100A16 IHC staining images. C: Normal lung (left) and bronchus (right). D: LUAD tissues. Image credit: Human Protein Atlas. Images were obtained from: v18.proteinatlas.org, via: http://www.proteinatlas.org/ENSG00000188643-S100A16/tissue/lung#img http://www.proteinatlas.org/ENSG00000188643-S100A16/tissue/bronchus#img http://www.proteinatlas.org/ENSG00000188643-S100A16/pathology/tissue/lung+cancer#img.

In TCGA-LUAD, 514 primary LUAD cases had RNA-seq and intact OS data recorded at the same time. By comparing *S100A16* expression using the RNA-Seq data, we found that the cancer tissues had significantly elevated *S100A16* expression compared with the normal lung tissues (N = 59) (Fig 1A and 1B). Using IHC staining images from the HPA, we further examined S100A16 protein expression in normal lung and LUAD tissues. Results showed that the pneumocytes in lung tissues had negative S100A16 staining (Fig 1C, left), but the respiratory epithelial cells in bronchus had moderate S100A16 expression (Fig 1C, right, red arrow). In comparison, the LUAD tissues had low to moderate S100A16 expression, in both cytoplasm and cell membrane (Fig 1D, red arrows).

### Comparison of *S100A16* expression between the groups with different clinicopathological parameters

**Fig 2:**
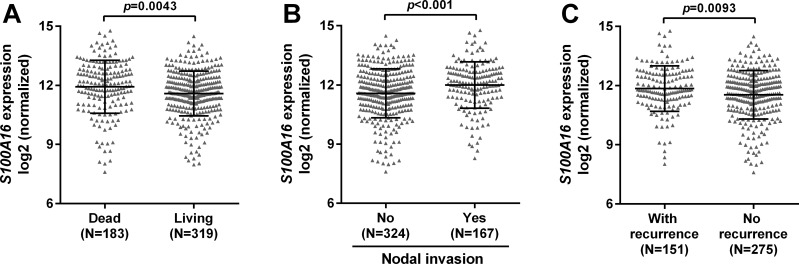
Comparison of *S100A16* expression between the groups with different clinicopathological parameters. **A-C.** Plots chart of *S100A16* expression between the living and dead cases (A), between the cases with or without nodal invasion (B) and between the patients with or without recurrence (C).

By examining *S100A16* expression between the living and dead cases, we found that the living cases had significantly lower *S100A16* expression compared with the deceased cases (*p* = 0.0043, Fig 2A). Then, we compared *S100A16* expression between the groups with different malignancies. Interestingly, we observed that the groups with nodal invasion and recurrence all had significantly higher *S100A16* expression compared with their respective counterparts (*p*<0.001 and *p* = 0.0093 respectively, Fig 2B and 2C).

### Aberrant *S100A16* expression was an independent prognostic indicator of unfavorable OS and RFS in LUAD patients

**Fig 3:**
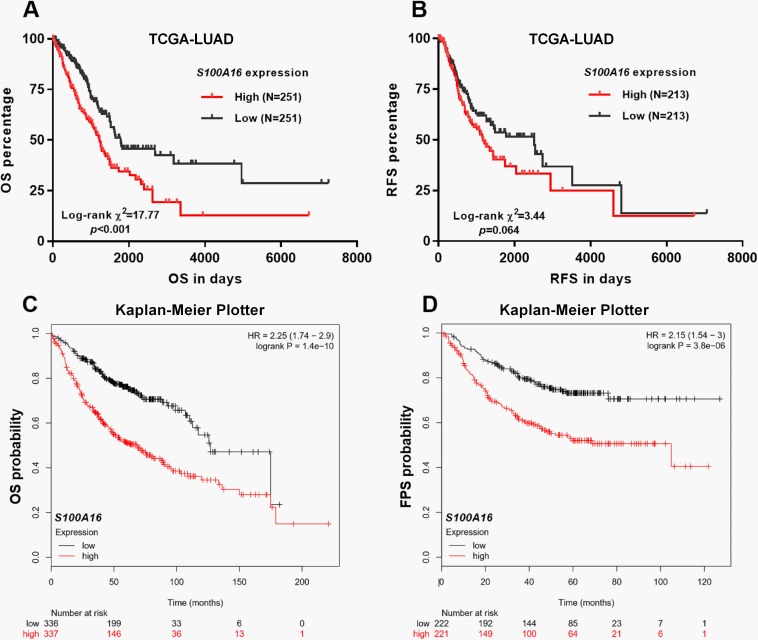
The association between *S100A16* expression and OS and RFS in LUAD patients. **A-B.** Kaplan-Meier curves of OS (A) and RFS (B) in LUAD patients, by using data from TCGA-LUAD. **C-D.** Kaplan-Meier curves of OS (C) and FPS (D) in LUAD patients. Results were generated by using Kaplan-Meier Plotter. Patients were grouped by the median *S100A6* expression.

By generating Kaplan-Meier curves of OS and RFS using survival data from TCGA-LUAD, we found that the group with high *S100A16* expression had significantly worse OS (*p*<0.001), and also had inferior RFS at the margin level of significance (*p* = 0.064) compared with the low *S100A16* expression group (Fig 3A and 3B). To verify these associations, we also conducted data mining in Kaplan-Meier Plotter. A pooled analysis based on 9 previous genomic data (GSE14814, GSE19188, GSE29013, GSE30219, GSE31210, GSE3141, GSE31908, GSE37745 and GSE50081) showed that high *S100A16* expression was significantly associated with worse OS (HR = 2.25, 95%CI: 1.74–2.90, *p*<0.001) (Fig 3C). A pooled result of five previous genomic data (GSE29013, GSE31210, GSE31908, GSE50081 and GSE8894) indicated that high *S100A16* expression was significantly associated with worse FPS (HR = 2.15, 95%CI: 1.54–3.00, *p*<0.001) (Fig 3D).

To investigate the independent prognostic value of *S100A16* expression in terms of OS and RFS in LUAD patients, we used the clinicopathological data and survival data in TCGA-LUAD. The 502 patients with primary LUAD were divided into high and low *S100A16* expression groups by the median gene expression. The association between *S100A16* expression and the clinicopathological parameters was summarized in Table 1.

**Table 1:** Comparison of the clinicopathological parameters between high and low *S100A16* expression groups.

Parameters		*S100A16* expression		χ^2^	*p* Value
		High (N = 251)	Low (N = 251)		
**Age (Mean ± SD)**		65.19 ± 10.01	65.45 ± 9.91		0.77
**Gender**	Female	129	142	1.36	0.24
	Male	122	109		
**Smoking History**	2/3/4/5	208	208	0.43	0.51
	1	33	39		
	Null	10	4		
**Pathologic Stage**	III/IV	66	40	7.85	0.005
	I/II	182	206		
	Discrepancy/null	3	5		
**Residual tumors**	R0	170	166	0.29	0.59
	R1/R2	7	9		
	RX/null	74	76		
**Radiation therapy**	No	185	203	3.16	0.076
	Yes	36	24		
	Null	30	24		
**Targeted molecular therapy**	No	130	166	0.47	0.0021
	Yes	89	61		
	Null	32	24		
**Recurrence status**	No	130	145	0.37	0.55
	Yes	76	75		
	Null	45	31		
**Living Status**	Living	142	177	10.53	0.0012
	Dead	109	74		

Smoking history: 1: lifelong non-smoker; 2: current smoker; 3. Current reformed smoker (for>15 yrs); 4. Current reformed smoker (for≤15 yrs); 5. Current reformed smoker (duration not specified); R0: No residual tumor; R1: Microscopic residual tumor; R2: Macroscopic residual tumor; RX: The presence of residual tumor cannot be assessed; null: no data.

Chi-square analysis showed that the high *S100A16* expression group had a significantly higher ratio of patients in advanced stages (III/IV) (66/248 *vs*. 40/246, *p* = 0.005) and death (109/251 *vs*. 74/251, *p* = 0.0012), compared with the low *S100A16* expression group (Table 1).

In univariate analysis, we found that advanced stages, the presence of residual tumors and increased *S100A16* expression were associated with unfavorable OS (Table 2). The following multiple variate analysis suggested that increased *S100A16* expression was an independent prognostic indicator of unfavorable OS (HR: 1.197, 95%CI: 1.050–1.364, *p* = 0.007, Table 2), after adjustment of pathologic stage, residual tumors and radiation therapy.

**Table 2:** Univariate and multivariate analysis of OS in LUAD patients.

Parameters	Univariate analysis				Multivariate analysis			
	*p*	HR	95%CI (lower/upper)		*p*	HR	95%CI (lower/upper)	
**Age** (Continuous)	0.330	1.008	0.992	1.023				
**Gender** Female *vs*. Male	0.670	0.939	0.702	1.256				
**Smoking history** 2/3/4/5 *vs*. 1	0.662	0.912	0.604	1.377				
**Pathologic Stage** III/IV *vs*. I/II	<0.001	2.646	1.942	3.606	<0.001	2.224	1.607	3.079
**Residual tumors** Yes *vs*. No	<0.001	3.937	2.204	7.033	0.002	2.522	1.390	4.578
**Radiation therapy** No *vs*. Yes	<0.001	0.478	0.328	0.696	0.047	0.664	0.444	0.995
**Targeted molecular therapy** No *vs*. Yes	0.309	0.843	0.606	1.172				
***S100A16* expression** (Continuous)	0.001	1.259	1.104	1.436	0.007	1.197	1.050	1.364

Then, by performing univariate and multivariate analysis in terms of RFS, we found that advanced stages, the presence of residual tumors and increased *S100A16* expression were associated with poor RFS in univariate analysis (Table 3). The following multiple variate analysis suggested that increased *S100A16* expression was an independent prognostic indicator of unfavorable RFS (HR: 1.206, 95%CI: 1.045–1.393, *p* = 0.011, Table 3), after adjustment of pathologic stage, residual tumors and radiation therapy.

**Table 3:** Univariate and multivariate analysis of RFS in LUAD patients.

Parameters	Univariate analysis				Multivariate analysis			
	*p*	HR	95%CI (lower/upper)		*p*	HR	95%CI (lower/upper)	
**Age** (Continuous)	0.323	1.008	0.992	1.025				
**Gender** Female *vs*. Male	0.574	1.097	0.794	1.516				
**Smoking history** 2/3/4/5 *vs*. 1	0.435	1.208	0.752	1.939				
**Pathologic Stage** III/IV *vs*. I/II	0.006	1.711	1.168	2.506	0.140	1.360	0.904	2.045
**Residual tumors** Yes *vs*. No	<0.001	3.808	1.838	7.892	0.008	2.782	1.312	5.899
**Radiation therapy** No *vs*. Yes	0.001	0.500	0.337	0.742	0.037	0.631	0.410	0.972
**Targeted molecular therapy** No *vs*. Yes	0.787	0.953	0.670	1.354				
***S100A16* expression** (Continuous)	0.002	1.262	1.092	1.458	0.011	1.206	1.045	1.393

### *S100A16* expression was negatively correlated with its DNA methylation in LUAD

**Fig 4:**
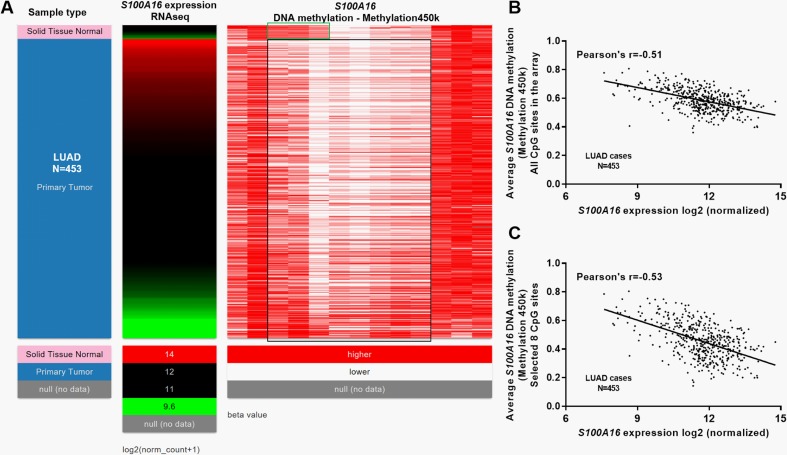
**The correlation between *S100A16* expression and its DNA methylation in LUAD tissues**. **A.** Heatmap of the correlation between *S100A16* expression and its DNA methylation. **B-C.** Regression analysis of the correlation between *S100A16* expression and the average methylation of all CpG sites in the array (B) or the selected 8 CpG sites (C).

**Fig 5:**
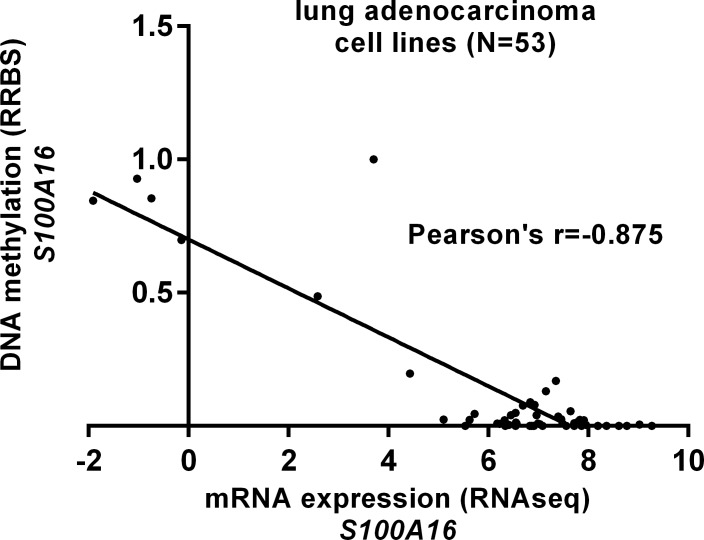
**The correlation between *S100A16* expression and its DNA methylation in 53 LUAD cell lines.** Regression analysis of the correlation between *S100A16* expression and its DNA methylation in 53 LUAD cell lines. RRBS: Reduced representation bisulfite sequencing.

**Fig 6:**
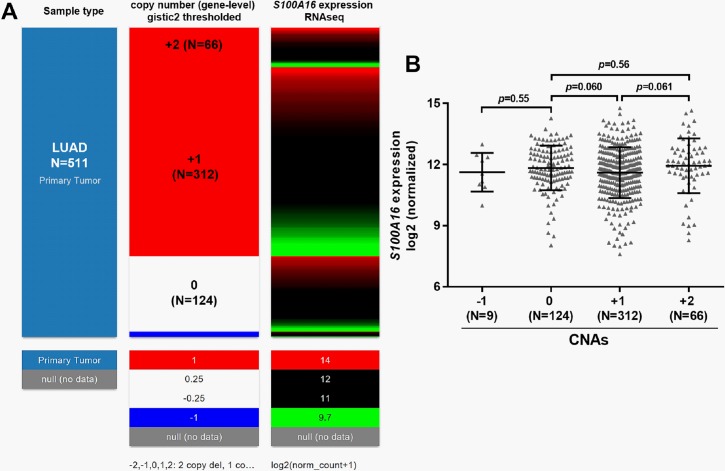
The association between *S100A16* expression and its CNAs. **A-B.** Heatmap (A) and plots chart (B) of *S100A16* DNA CNAs and its RNA expression. -2: homozygous deletion; -1: heterozygous loss, 0: copy-neutral; +1: low-level copy gain; +2: high-level amplification.

DNA methylation is a common epigenetic mechanism influencing gene expression in lung cancer [17]. By examining the DNA methylation and RNA expression data in TCGA-LUAD, we found that some CpG sites were generally hypermethylated in normal tissues, but not in LUAD tissues (Fig 4A, green box). 453 primary cases had *S100A16* DNA methylation and RNA expression measured at the same time (Fig 4A). The heatmap suggested that *S100A16* expression might be negatively correlated with the methylation of some CpG sites (Fig 4A, black box). Then, we performed regression analysis to evaluate the correlation between *S100A16* expression and the average methylation of all CpG sites in the array or the selected 8 CpG sites (Fig 4A, black box). The regression analysis confirmed a moderate negative correlation between *S100A16* expression and its DNA methylation, no matter by using the average methylation of all CpG sites in the array (Pearson’s r = -0.51, Fig 4B) or the selected 8 CpG sites (Pearson’s r = -0.53, Fig 4C). Then, we examined *S100A16* expression and its DNA methylation in 53 lung adenocarcinoma cell lines, by using data from the CCLE. Regression analysis confirmed a strong negative correlation Pearson’s r = -0.875) between *S100A16* expression and its DNA methylation in the cell lines (S2 Table and Fig 5). Then, we examined the association between *S100A16* DNA CNAs and its expression. In 511 LUAD patients with CNA measured, 66 (12.9%) cases had high-level amplification (+2), 312 cases (61.1%) had low-level copy gain (+1) (Fig 6A). However, these DNA amplifications might not influence *S100A16* expression (Fig 6B).

## Discussion

In the past years, a series of secondary analyses using data in TCGA have identified some promising and potential biomarkers in lung cancer. For example, checkpoint with fork-head and ringfinger domains (*CHFR*) expression is a valuable predictor for response to taxane-based first-line chemotherapy in NSCLC [18]. One recent study using data in TCGA found that *CHFR* expression was associated with epidermal growth factor receptor (*EGFR*) exon19/21 mutations in adenocarcinoma and male gender in squamous cell carcinoma [18]. Another recent study assessed the prognostic value of epithelial cell transforming 2 (*ECT2*) in NSCLC and observed that increased *ECT2* expression might independently predict poor OS and RFS in LUAD, but not in lung squamous cell carcinoma [17]. Using the same data cohort, another study found that Secreted frizzled-related protein 3 (*SFRP3*), a putative tumor suppressor in LUAD, is epigenetically silenced and is associated with poor prognosis [19].

In this study, using RNA-seq data in TCGA-LUAD, we confirmed significantly upregulated *S100A16* in LUAD than in normal lung tissues. By setting median *S100A16* expression as the cutoff, we found that high *S100A16* expression was correlated with inferior OS. Interestingly, although the RFS curves using data from TCGA-LUAD failed to identify significant association between *S100A16* expression and RFS, pooled results of previous genomic studies suggest that high *S100A16* expression might influence RFS. Therefore, we hypothesized that the cutoff setting might affect the results. Thus, *S100A16* expression was set as a continuous variable in COX survival analysis. The following univariate and multivariate analysis confirmed the independent prognostic value of S*100A16* expression in terms of OS, which is consistent with the findings in two recent studies [7, 8]. In addition, we also found that increased *S100A16* expression was an independent prognostic indicator of unfavorable RFS (HR: 1.206, 95%CI: 1.045–1.393, *p* = 0.011), after adjustment of pathologic stage and residual tumors. Therefore, we infer that *S100A16* expression might be a potential biomarker of unfavorable OS and RFS in LUAD patients.

One previous study found that S100A16 could promote EMT in breast cancer cells by elevating the expression of transcription factors Notch1, ZEB1, and ZEB2 [3]. In fact, EMT is an important mechanism leading to facilitated lymph node invasion and metastasis of LUAD, as well as chemoresistance and radioresistance [20–23]. In addition, EMT is also inversely associated with T-cell infiltration in NSCLC, which results in poor response to immunotherapy [24]. These mechanisms help to explain the association between *S100A16* expression and poor survival in LUAD patients. However, the detailed mechanisms should be explored in the future.

The mechanisms of gene dysregulations are quite complex in NSCLC. Both genetic and epigenetic alterations can influence gene expression and modulate cancer cell behaviors, such as DNA amplification and methylation. For example, *ECT2* DNA amplification is common in invasive LUAD [17, 25]. *HER2* gene amplification and *SPTBN1-ALK* gene fusion may drive crizotinib resistance in LUAD [26]. De novo *ERBB2* amplification could induce intrinsic resistance to erlotinib in *EGFR-L858R* mutated TKI-naive LUAD [27]. Hypermethylation mediated downregulation of runt-related transcription factor 3 (*RUNX3*) could induce docetaxel chemoresistance in LUAD [28]. *CDH13* promoter is hypermethylated in LUAD and might be a potential diagnostic biomarker for diagnosis [29]. The heparan sulfate 6-O-endosulfatase (*SULF2*) expression in NSCLC is negatively modulated by its DNA methylation in NSCLC. Suppressed SULF2 expression could increase the expression of INF-inducible regulator of ubiquitination (ISG-15), which is a marker for increased sensitivity to topoisomerase-1 inhibitors such as camptothecin (CPT) [30]. In this study, we examined the effect of DNA methylation on *S100A16* expression in LUAD patients and in 53 LUAD cell lines. Regression analysis confirmed a significant negative correlation between *S100A16* expression and its DNA methylation, suggesting that DNA methylation might be an important mechanism influencing *S100A16* expression in LUAD. Besides, we also examined the DNA amplification status of *S100A16*. Results showed that although DNA amplification (+1/+2) was frequent (378/511, 74%) in LUAD patients, it was not associated with increased *S100A16* expression.

## Conclusion

Based on findings above, we infer that aberrant *S100A16* expression might be modulated by its DNA hypomethylation and serves as an independent prognostic indicator of unfavorable OS and RFS in LUAD.

## Supporting information

S1 Table Level-3 data in TCGA-LUAD downloaded for secondary analysis. (XLSX)

S2 Table *S100A16* expression (RNA-seq) and DNA methylation in 53 LUAD cell lines. (XLSX)
